# The Compatibility of the *Epichloë bromicola*–*Hordeum* Association

**DOI:** 10.3390/jof12010053

**Published:** 2026-01-11

**Authors:** Jing Liu, Jiping Li, Tao Li, Zhengfeng Wang, Chunjie Li

**Affiliations:** 1 State Key Laboratory of Herbage Improvement and Grassland Agro-Ecosystems, Lanzhou University, Lanzhou 730000, China; liujing2024@swust.edu.cn (J.L.); lijp21@lzu.edu.cn (J.L.); litao@imun.edu.cn (T.L.); 2 Key Laboratory of Grassland Livestock Industry Innovation, Ministry of Agriculture and Rural Affairs, Lanzhou 730000, China; 3 Engineering Research Center of Grassland Industry, Ministry of Education, Gansu Tech Innovation Centre of Western China Grassland Industry, Lanzhou 730000, China; 4 College of Pastoral Agriculture Scienceand Technology, Lanzhou University, Lanzhou 730000, China; 5 College of Life Sciences and Agri-Forestry, Southwest University of Science and Technology, Mianyang 621010, China; 6 Chongqing Wanzhou Airport Co., Ltd., Chongqing 404100, China; 7 College of Grassland, Inner Mongolia Minzu University, Tongliao 028000, China; 8 Institute of Plant Protection, Gansu Academy of Agricultural Science, Lanzhou 730070, China; wangzhf1006@163.com

**Keywords:** colonization pattern, compatibility, hyphae vacuolization, hyphal concentration, siderophore gene

## Abstract

Background: Artificial inoculation of *Epichloë* endophytes into elite forage germplasm aims to establish beneficial symbioses for developing high-yield, high-quality, and stress-tolerant cultivars, but host specificity of the fungi often causes compatibility issues in non-natural hosts. Methods: The *E. bromicola* isolated from native wild barley was inoculated into cultivated wild barley (*Hordeum brevisubulatum*) and cultivated barley (*Hordeum valgare*), forming Hb+Eb and Hv+Eb. The NHb+Eb (native wild barley naturally infected with *E. bromicola*) served as a control. We analyzed fungal colonization patterns and symbiotic gene regulation to clarify the compatibility between *E. bromicola* and non-natural hosts. Results: Compared with NHb+Eb and Hb+Eb, *E. bromicola* in Hv+Eb showed obvious hyphal vacuolization. *E. bromicola* colonization altered host trichome morphology and induced stomatal closure. Correspondingly, expression of the siderophore biosynthesis gene *sidN* and the NADPH oxidase complex genes (*NoxA*, *NoxB*, *NoxR*, *RacA*) was significantly lower (*p* < 0.05) in Hv+Eb than in Hb+Eb and NHb+Eb. Conclusions: This study reveals that the incompatibility between cultivated barley and *E. bromicola* is characterized by altered hyphal morphology, which is linked to the downregulation of *sidN* and *Nox*. These findings provide a critical theoretical foundation for developing highly compatible cereal-*Epichloë* germplasms.

## 1. Introduction

*Epichloë* endophytes form mutualistic symbioses with grasses [1]. The host grass provides nutrients and habitat for fungal growth and transmission [2]. In return, the *Epichloë* endophytes synthesize alkaloids such as peramine and loline, which confer insect resistance to the host [3,4]. Additionally, these symbiotic fungi enhance host tolerance to abiotic stresses and promote grass growth, thereby reinforcing the stability and benefits of the association [5]. Therefore, screening non-toxic strains that do not produce alkaloids harmful to livestock and developing novel grass–endophyte symbiotic associations through artificial inoculation hold significant promise for improving forage quality while ensuring animal safety [6]. Internationally, breeding efforts utilizing *Epichloë* endophytes have primarily focused on a few forage species, notably perennial ryegrass (*Lolium perenne*) and tall fescue (*Festuca arundinacea*), with several commercial varieties such as MaxQ, MaxQ II [7], and Avanex [8] already successfully marketed. In contrast, the development of symbiotic associations between *Epichloë* endophytes and important cereal crops remains under active research [7,8,9].

The *Epichloë* endophyte AR3068, isolated from *Elymus dahuricus*, was inoculated into two rye (*Secale cereale*). This resulted in divergent outcomes: one symbiont exhibited severe stunting, while the other resembled healthy uninoculated plants. In a parallel experiment, the *Epichloë* endophyte AR3060 isolated from *E. dahuricus* was inoculated into wheat (*Triticum aestivum*), and the associations were poorly developed or dwarfed [8,9]. The *Epichloë bromicola* isolated from wild barley (*Hordeum brevisubulatum*) was inoculated into cultivated barley *Hordeum vulgare* cv. Yangsimai No. 1 and *H. vulgare* var. nudum cv. Chaiqing No. 1, resulting in the formation of two novel associations: Lan Da No. 1 and Lan Qing No. 1. Compared with Lan Qing No. 1, the growth period of Lan Da No. 1 was 5 days earlier [6]. The observed phenotypic variation in these associations demonstrates that novel symbiotic relationships can be established by inoculating *Epichloë* endophytes into closely related, non-natural hosts [7]. Conversely, this process may also trigger host-specific recognition, leading to compatibility issues between *Epichloë* and its new host [10]. These compatibility problems include host dysplasia [8], changes in mycelial ultrastructure, mycelial colonization patterns, and premature mycelial death [11,12]. Such disturbances encompass reduced hyphal biomass, degeneration of crystalline structures, and thinning of the cell wall [12]. Furthermore, fungal colonization within the vascular bundles may disrupt phloem and xylem transport, leading to host growth inhibition and developmental defects [13]. Despite the critical role of fungal colonization and morphology in determining symbiont compatibility, little is known about the compatibility of *E. bromicola* with non-natural hosts like barley.

As obligate grass endophytes, *Epichloë* endophytes depend entirely on host apoplast nutrients for successful colonization and survival [14]. Among these, iron has proven essential for maintaining both the *E. festucae*–*Lolium perenne* and *E. festucae* symbiosis and fungal iron homeostasis [15]. Siderophores produced by *Epichloë* endophytes are essential for acquiring iron from the host, enabling successful colonization, survival, and vertical transmission [16,17]. Without this siderophore-mediated iron uptake, the mutualistic symbiosis would collapse [15]. *Epichloë* endophytes employ specialized systems to acquire iron from iron-limiting environments, including high-affinity iron-chelating molecules (siderophores) and/or membrane-bound reductive iron assimilation (RIA) systems [18]. Siderophores are small chelator molecules with extremely high affinity for ferric (Fe^3+^) iron, including extracellular (secreted) siderophores and intracellular siderophores. In *E. festucae*, the extracellular siderophore *epichloënin A* (EA) is synthesized by *SidN* for iron uptake. while *SidC* produces the intracellular siderophore ferricrocin (FC) for iron storage, sequestration, and transport [17]. Genomic studies in *E. festuca* have further identified key components of the RIA system (*FetC*, *FtrA*, *SreA*, and *HapX*) [19,20], which has been functionally characterized as a high-affinity mechanism essential for vegetative growth [16]. Iron deficiency impairs partner metabolism and destabilizes the symbiosis, whereas iron overload induces oxidative stress via Fenton-generated ROS, similarly disrupting the symbiotic balance [21]. In filamentous fungi, an intact NADPH oxidase complex (comprising *NoxA*, *NoxB*, *NoxR*, and *RacA*) is essential for sustaining mutualistic symbiosis, whereas its destruction promotes a pathogenic outcome rather than mutualistic symbiosis [22,23,24]. In contrast, while the high-affinity iron uptake system is established as essential for endophyte colonization in natural symbiotic associations, current research on its role in maintaining compatibility remains limited and predominantly focused on the *E. festucae*–perennial ryegrass model. The function of this system in artificially established novel associations, particularly with cereal crops, remains largely unexplored. Investigating the role of the iron uptake system in *Epichloë* endophytes is crucial for understanding and improving compatibility in these novel symbiotic partnerships.

In this study, the *Epichloë bromicola* isolated from native wild barley (*Hordeum brevisubulatum*) was inoculated into cultivated wild barley and cultivated barley (*Hordeum valgare*), forming Hb+Eb (cultivated wild barley inoculated with *E. bromicola*) and Hv+Eb (cultivated barley inoculated with *E. bromicola*). The NHb+Eb (native wild barley naturally infected with *E. bromicola*) served as a control. To identify compatibility barriers in non-natural hosts, fungal colonization patterns in the three hosts were examined using light, transmission electron, and scanning electron microscopy. Gene cloning and expression analysis were further employed to assess the role of symbiotic gene regulation in compatibility. In summary, this work aims to elucidate the key factors affecting the *E. bromicola*–*Hordeum* symbiosis, providing a foundation for breeding forage germplasm with high yield, superior quality, and stress tolerance.

## 2. Materials and Methods

### 2.1. Strain

*Epichloë bromicola* strain WBE1, isolated from wild barley, was provided by the College of Pastoral Agriculture Science and Technology, Lanzhou University, China. It can produce peramine and chanoclavine I, but not loline, lolitrem B, and complex ergot alkaloids [25]. Peramine is an insect-deterrent alkaloid.

### 2.2. Plant Material

The cultivated barley of Yangsimai No. 1 (*Hordeum vulgare* cv. Yangsimai No. 1) was purchased from Jiangsu Suqian Tijing Landscaping Engineering Co., Ltd. The cultivated wild barley seeds uninfected with *E. bromicola* were collected from the experimental field of Jinniu Mountain in Yuzhong, College of Grassland Agricultural Science and Technology, Lanzhou University. The seeds of native wild barley (*Hordeum brevisubulatum*), both naturally infected and uninfected with *E. bromicola*, were collected from their natural habitat. These seeds were stained with aniline blue to observe the infection state under the microscope. A spike was considered infected if at least one of the ten examined seeds tested positive for infection under a light microscope (100×) using the squash technique. In brief, caryopses that had been pre-incubated in NaOH solution for a minimum of 24 h were squashed onto a cover slip and stained with aniline blue. This method enabled the differential identification of fungal hyphae from the aleurone layer and seed coat. Seeds from native wild barley naturally infected with *E. bromicola*, native wild barley uninfected with *E. bromicola*, and cultivated wild barley uninoculated with *E. bromicola* were stored in a 4 °C refrigerator for subsequent experiments [5].

### 2.3. Reagents

The chemical reagents were purchased from Sinopharm Chemical Reagent Co., Ltd. The molecular reagents were purchased from Sangon Biotech (Shanghai) Co., Ltd. The murashige and skoog (MS) medium was purchased from Hangzhou Best Biotech Co., Ltd.

### 2.4. Construction of E. bromicola–Hordeum Association

The experiment was conducted in a greenhouse at Lanzhou University’s Yuzhong. A sterilized seedling wound inoculation protocol was employed, modified from established methods [6,26]. The healthy and plump seeds of native wild barley naturally infected with *E. bromicola*, native wild barley uninfected with *E. bromicola*, cultivated wild barley uninfected with *E. bromicola,* and cultivated barley were selected. The seeds were surface sterilized by immersion in 75% (*v*/*v*) ethanol solution with agitation on a shaker for 5 min, followed by five rinses with sterile water. Subsequently, the seeds were treated with 10% (*v*/*v*) NaClO solution with agitation for 5 min and rinsed five times with sterile water. After sterilization, the seeds were air-dried in the dark and placed on MS medium (pH 5.8) for germination under dark conditions (the seeds of native wild barley naturally infected with *E. bromicola*, native wild barley uninfected with *E. bromicola*, and cultivated wild barley inoculated with *E. bromicola* were cultured for 5–6 days, while cultivated barley seeds were cultured for 3–4 days). Once germinated, the seedlings were transferred to a growth chamber set at 22 ± 1 °C, 75% humidity, and a 24 h photoperiod for further growth. Throughout the process, any seeds showing fungal or bacterial contamination were removed.

Subsequently, when the axenic seedlings of cultivated wild barley uninoculated with *E. bromicola* had developed 3–4 leaves and reached a height of 4–5 cm, and the cultivated barley axenic seedlings had developed 2 leaves and reached a height of 8–10 cm, they were transferred to a laminar flow hood for inoculation. The cultivated wild barley uninoculated with *E. bromicola* and cultivated barley seedlings were inoculated by making an incision near the coleoptile tissue of the axenic seedlings, followed by inserting *Epichloë* mycelia with medium into the incised area. As a result, cultivated wild barley inoculated with *E. bromicola* association (Hb+Eb) and cultivated barley inoculated with *E. bromicola* association (Hv+Eb) were constructed. Control seedlings underwent the same wounding procedure but received a plug of sterile ½ MS medium alone, without *Epichloë* mycelium. As a result, cultivated wild barley uninoculated with *E. bromicola* (Hb) and cultivated barley uninoculated with *E. bromicola* (Hv) were constructed, which served as negative controls of Hb+Eb and Hv+Eb, respectively. In parallel, seedlings of the native wild barley naturally infected with *E. bromicola* and native wild barley uninfected with *E. bromicola* were also inoculated with PDA medium alone, which, designated as NHb+Eb and NHb, served as the positive control of both Hb+Eb and Hv+Eb. The NHb is the negative control of NHb+Eb.

All treated seedlings were maintained in a growth chamber at 24 °C under a 12/12 h light/dark cycle for 5 days. Surviving seedlings were subsequently transplanted into pots (14 cm diameter × 16 cm depth) containing sterilized vermiculite. The pots were randomly arranged in a variable-temperature greenhouse set to a 16/8 h light/dark cycle, 25/20 °C day/night temperatures, 800 μmol m^−2^s^−1^ light intensity, and 60% relative humidity. Plants were regularly irrigated with Hoagland’s nutrient solution.

NHb+Eb: native wild barley (*H. brevisubulatum*) naturally infected with *E. bromicola.*

NHb: native wild barley uninfected with *E. bromicola*.

Hb+Eb: cultivated wild barley inoculated with *E. bromicola*.

Hb: cultivated wild barley uninoculated with *E. bromicola*.

Hv+Eb: cultivated barley (*H. vulgare*) inoculated with *E. bromicola*.

Hv: cultivated barley uninoculated with *E. bromicola*.

### 2.5. Colonization Detection of E. bromicola in Barley

#### 2.5.1. Molecular Technique

Protocol for DNA Extraction from *E. bromicola*: *E. bromicola* strains were purified on potato dextrose agar (PDA) medium. After four weeks of incubation, four 1 mm mycelial plugs were punched from the margin of the mycelial colony using a sterile cork borer. These plugs were inoculated into 500 mL flasks containing 250 mL of potato dextrose broth (PDB) medium, followed by incubation on a shaker at 190 rpm for 7 days. The mycelia were harvested by filtration through sterile gauze, blotted dry with sterile filter paper, and subsequently lyophilized in a freeze dryer. Genomic DNA of the endophytic fungus was extracted using the D3195 HP Kit (Omega, Beijing, China) according to the manufacturer’s instructions, and the extracted DNA was stored at −20 °C.

Protocol for DNA Extraction from *E. bromicola*–*Hordeum* Association: The leaf samples were collected from the inoculated plants of NHb, Hb+Eb, and Hv+Eb for genomic DNA extraction, using the sodium dodecyl sulfate (SDS) method. The procedure was performed as follows: approximately 0.5 g of fresh leaf tissue was placed in a 1.5 mL tube containing steel beads, flash-frozen in liquid nitrogen, and ground thoroughly using a bead mill. Then, 450 μL of preheated DNA extraction buffer (65 °C) was added, followed by vortexing for 20 s and incubation in a 65 °C water bath for 20 min to ensure complete cell lysis. Subsequently, 315 μL of chloroform–isoamyl alcohol (24:1, *v*/*v*) was added, and the mixture was vortexed for 30 s, left to stand for 5 min, and centrifuged at 14,000 rpm for 5 min at 4 °C. A 300 μL aliquot of the supernatant was transferred to a new tube, mixed with 210 μL of ice-cold isopropanol, vortexed, incubated for 5 min, and centrifuged again under the same conditions. The supernatant was discarded, and the pellet was gently washed three times with 500 μL of 75% ethanol, with brief centrifugation between washes. After drying, the DNA pellet was dissolved in 1/10 TE buffer containing 20 μg/mL RNase A. The purity and concentration of DNA were assessed using a Promega NanoDrop spectrophotometer (Promega Corporation, Madison, WI, USA), and all samples were uniformly diluted to 40 ng/μL for subsequent use.

PCR Amplification of *E. bromicola* in *Hordeum*: To verify the colonization status of the *Epichloë* endophyte, PCR amplification was performed on the extracted DNA using gene-specific primers targeting the *Epichloë* housekeeping genes *tefA* and *tubB*, as well as the peramine synthase gene (*perA*). The primers were *tefA*-exon 1d-1: GGG TAAG GAC GAA AAG ACT CA; *tefA*-exon 5u-1: CGG CAG CGA TAA TCA GGA TAG [27]; *tubB*-exon 1d-1: GAG AAA ATG CGT GAG ATT GT; *tubB*-exon 4u-2: GTT TCG TCC GAG TTC TCG AC [28]; *perA2*-F: CGT CGT GGT AAC GCA CGC AAA CG; and *perA2*-R: CAG TCT GCC TTG CCG ACC GGG GT [29]. The cycling conditions for PCR were adapted from Chen et al. (2019) [25]. PCR amplification was performed in a 25 µL reaction system, which consisted of 12.5 µL of 2× Taq MasterMix (ComWin Biotech Corp., Ltd., Beijing, China; containing 1.0 U Taq DNA polymerase, 1.5 mM MgCl_2_, and 200 µM of each dNTP), 9.5 µL of double-distilled H_2_O, 10 ng of DNA template, and 1 µM each of the gene-specific forward and reverse primers. Distinct thermal cycling profiles were applied for different target genes. For the amplification of *tef1* and *tub2*, the procedure was set as follows: an initial denaturation step at 94 °C for 5 min; 30 cycles of denaturation at 94 °C for 30 s, annealing at 55 °C (*tefA*) or 53 °C (*tubB*) for 45 s, and extension at 72 °C for 1 min; and a final extension step at 72 °C for 10 min. In contrast, the amplification of *perA2* adopted the following cycling parameters: initial denaturation at 94 °C for 1 min, 30 cycles of denaturation at 94 °C for 15 s, annealing at 56 °C for 30 s, and extension at 72 °C for 15 s, with a final extension step at 72 °C for 10 min.

*E*. *bromicola* strain was used as a positive control template, and sterile water was used as a negative control template. All PCR products were detected by 1.2% agarose gel electrophoresis and observed under ultraviolet light. The gel images were taken using the ChampGel 6000 gel imager (SINSAGE SCI-TECH CO., LTD., Beijing, China).

Following molecular identification and confirmation, the seedlings inoculated with *E. bromicola* but not successfully infected were excluded, and the seedlings infected with *E. bromicola* were selected (NHb+Eb, Hb+Eb, Hv+Eb) for subsequent experiments.

#### 2.5.2. Light Microscope

Sampling was performed when the plants reached the seedling and tillering stages. The upper, middle, and lower segments of the leaf sheaths from the NHb+Eb, Hb+Eb, and Hv+Eb associations were stained with aniline blue to determine the endophyte colonization status. Sampling and staining were conducted following Liu et al. (2025) [5]. The specific procedure was as follows: First, the leaf sheath epidermis was gently scraped with a scalpel. Then, the samples were stained with 1% aniline blue solution (containing 10 mL lactic acid, 10 mL glycerol, and 10 mL phenol, made up to 80 mL with distilled water). The younger leaves were left stationary for a brief period, while older leaves were briefly heated over an alcohol lamp flame. Finally, fungal presence in NHb+Eb, Hv+Eb, and Hb+Eb associations was examined and recorded under an Olympus optical microscope (Tokyo, Japan). The aniline blue-stained images were observed using a bright-field microscope.

The colonized leaf samples described above were collected and immediately soaked in 2.5% glutaraldehyde for primary fixation. Subsequently, 10 samples were selected separately from each of the NHb+Eb, Hb+Eb, and Hv+Eb groups for subsequent observation and analysis via transmission electron microscopy (TEM) and scanning electron microscopy (SEM).

#### 2.5.3. The Transmission Electron Microscope

The leaves infected with *E. bromicola* (NHb+Eb, Hb+Eb, and Hv+Eb) and uninfected/uninoculated with *E. bromicola* (NHb, Hb, and Hv) at the seedling stage and tillering stage were cut with a double-edged knife to 1 × 3 × 1 mm^3^ and fixed in 2.5% glutaraldehyde at room temperature for 24 h. The preparation procedure generally followed the standard TEM protocol described by Rahnama et al. (2018) [29] with minor modifications. The sampling was rinsed with phosphate buffer (pH 7.0) three times and then fixed with 1% osmium tetroxide phosphate buffer at 4 °C for 2 h. The fixed samples were dehydrated with ethanol and embedded in resin for sectioning. The sample was cut into extremely thin sections (70–90 nm) using a LeciaEMU C7 ultra-thin slicing machine (Leica Microsystems GmbH, Wetzlar, Germany) with a diamond knife. Lastly, the sections were stained with 0.5% acetic acid dioxygen axis and lead citrate and then observed using a Tecnai G2 Spirit Bio–TWIN 120 KV (FEI company, Hillsboro, OR, USA) transmission electron microscope.

#### 2.5.4. The Scanning Electron Microscope

The leaves infected with *E. bromicola* (NHb+Eb, Hb+Eb, and Hv+Eb) and uninfected/uninoculated with *E. bromicola* (NHb, Hb, and Hv) at the seedling stage and tillering stage were cut with a double-edged knife to 3–5 mm and soaked in glutaraldehyde fixative for 20–30 min. Secondly, the samples were soaked in 60%, 70%, 80%, 90%, and 100% ethanol to remove water, 15–20 min each time. Then, the sample was soaked in tert-butanol for 15–20 min to remove ethanol. If tert-butanol was a solid, it should be placed at room temperature and melted at 26 °C. Finally, the above-treated samples were refrigerated to a solid state. The freeze-dried samples were bonded to the sample stage with conductive adhesive and sprayed with gold for 15 s. The morphology was observed by JEOL JSM-IT200 InTouchScope™ Scanning Electron Microscope (Akishima, Tokyo, Japan) with a voltage of 10. The sample preparation procedure generally followed the standard SEM protocol described by Venkatesh et al. (2018) [30], with minor modifications.

#### 2.5.5. Measurement of the Plant Structure

Leaves were randomly selected from plants of both the *E. bromicola*-infected/colonized groups (NHb+Eb, Hb+Eb, Hv+Eb) and the *E. bromicola*-uninfected/uncolonized groups (NHb, Hb, Hv) at the seedling and tillering stages. There were five biological replicates per group, each with three technical replicates. The cell wall thickness, trichome size and stomatal size of the leaves, and mycelial diameter were measured using ImageJ software (https://imagej.nih.gov/ij/).

### 2.6. The Gene Cloning

#### 2.6.1. Primer Design and Specificity Validation

By reading the literature from NCBI (https://www.ncbi.nlm.nih.gov), the genes related to the mutualism of endophytic fungi and hosts were queried, and their primers or gene sequences were found. The universal primers were designed by Sangon Biotech (Shanghai) Co., Ltd. (Shanghai, China) or NCBI and synthesized by Sangon Biotech (Shanghai) Co., Ltd. (Table 1).

**Figure 1:**
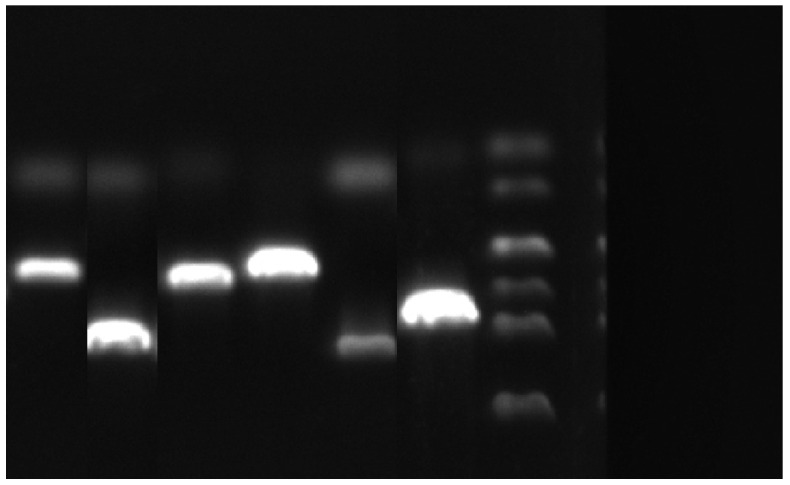
Verification of symbiotic genes of *E. bromicola*. Note: From left to right are *NoxB*, *NoxA*, *Sid N*, *RacA*, *NoxR*, and *PerA*, respectively.

Using genomic DNA of the *E. bromicola* as the template, the PCR reaction system was identical to that described in Section 2.5.1. Upon completion of PCR amplification, 5 μL of the PCR product was analyzed via 1.2% agarose gel electrophoresis. Gel images were captured using a ChampGel 6000 gel documentation system (SINSAGE, Beijing, China) to obtain the target bands (Figure 1). The target genes were recovered using the Tiangen Agarose Gel DNA Extraction Kit (TIANGEN BIOTECH (BEIJING) CO., LTD., Beijing, China), with the procedures performed strictly following the manufacturer’s instructions. An appropriate amount of the recovered gene products was subjected to electrophoresis to evaluate the recovery efficiency.

#### 2.6.2. Vector Construction and Preparation of Competent Cells

The ligation of the target gene and vector was performed using the pGEM-T^®^ Easy Vector (Sangon Biotech, Shanghai, China) with a total reaction volume of 10 μL, strictly following the manufacturer’s instructions. The ligation product was then transformed into JM109 competent cells according to the following procedure:

(1) LB solid plates containing ampicillin, IPTG, and X-Gal were prepared.
(2) The ligation mixture was briefly centrifuged (30 s, 6000 r/min), and 2 μL was transferred into a sterile 1.5 mL microcentrifuge tube placed on ice.
(3) JM109 competent cells were thawed on ice and gently mixed by flicking the tube.
(4) A 50 μL aliquot of the JM109 competent cells was carefully added to the tube containing the ligation product and mixed gently.
(5) The mixture was heat-shocked in a 42 °C water bath for 45–50 s, then immediately placed on ice for 2 min to terminate the reaction.
(6) A volume of 800 μL of LB liquid medium (without ampicillin) was added to each tube, followed by incubation in a shaker at 37 °C with shaking at 150 rpm for 1.5 h.
(7) After incubation, the cultures were centrifuged at 6000 rpm for 5 min at room temperature. Approximately 400 μL of the supernatant was discarded, and the pellet was resuspended in the remaining medium.
(8) A 100 μL aliquot of the resuspended cells was evenly spread onto LB plates containing ampicillin, IPTG, and X-Gal.
(9) Once the liquid had been completely absorbed, the plates were inverted and incubated overnight at 37 °C. Colony growth and blue–white screening results were observed the following day.

#### 2.6.3. Protocol for Recombinant Plasmid Construction, Restriction Digestion, and Verification

**Figure 2:**
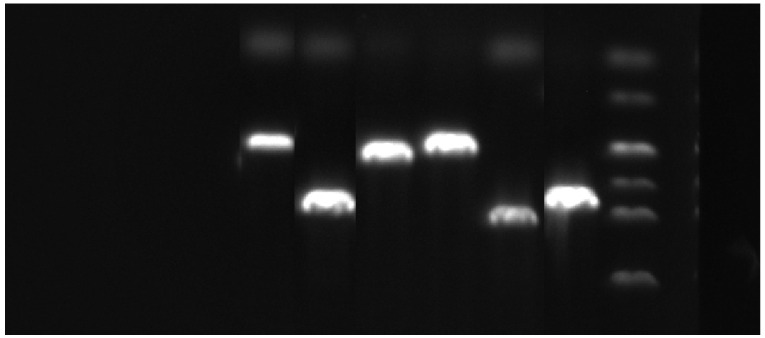
Verification of symbiotic genes of *E. bromicola*. Note: From left to right are *NoxB*, *NoxA*, *Sid N*, *RacA*, *NoxR*, and *PerA*, respectively.

White single colonies were randomly selected from the screening plates and inoculated into two separate culture volumes: 700 µL of LB medium (with ampicillin) for initial growth and 10 mL of the same medium for subsequent plasmid preparation. Both cultures were incubated overnight at 37 °C with shaking at 150 rpm. The smaller culture (700 µL) was used directly as the template for PCR verification with universal primers (Figure 2). Positive clones identified by electrophoresis were then subjected to sequencing. Based on the confirmed sequences, specific primers were designed (Sangon Biotech, Table 2).

For the expanded 10 mL culture, plasmids were extracted using the TianPrep High Purity Plasmid Mini Kit (Tiangen) according to the manufacturer’s instructions. The purified plasmid DNA was dissolved in 40 μL of TE buffer and further verified by PCR and electrophoretic analysis. Finally, based on the vector map, the circular plasmid was linearized with the ApaL I restriction enzyme using a corresponding digestion kit to complete the preparation of the insert-ready construct.

### 2.7. Analysis of Symbiotic Gene Expression in E. bromicola–Hordeum Association

#### 2.7.1. Protocol for RNA Extraction from *E. bromicola*–Hordeum Association

Leaf samples were collected at both the seedling and tillering stages from three associations: naturally *E. bromicola*-infected wild barley (NHb+Eb), *E. bromicola*-inoculated cultivated wild barley (Hb+Eb), and *E. bromicola*-inoculated cultivated barley (Hv+Eb). We prepared three biological replicates per group, each with three technical replicates. Total RNA was extracted from the leaves using the Total RNA Extractor (Trizol) kit (GENSTONE BIOTECH (BEIJING) CO., LTD., Beijing, China). The RNA samples were treated with DNase I to remove genomic DNA contamination. RNA purity and concentration were measured with the Qubit RNA Assay Kit (Thermo Fisher Scientific, Waltham, MA, USA), and integrity was assessed using the Agilent Bioanalyzer 2100 system (Agilent Technologies, Carpinteria, CA, USA).

#### 2.7.2. Relative Quantification of Symbiotic Gene Expression in *E. bromicola*–Hordeum Association

Following RNA extraction, total RNA from the 9 transcript samples was diluted to a uniform concentration of 100 ng/μL using DNase-free H_2_O. Subsequently, single-stranded cDNA was synthesized from the diluted RNA using the Toloscript RT EosvMix Kit (TOLOBIO, Shanghai, China) for qPCR (with 2-step gDNA Erase-Out, #22106). RT-PCR was performed on a Roche LightCycler^®^ 480 instrument using TB Green^®^ Premix Ex Taq™ II (Takara Bio Inc., Otsu, Japan). The reaction mixture (10 μL total volume) consisted of 5 μL of TB Green Premix Ex Taq II, 0.5 μL each of forward and reverse primers (10 μM), 1 μL of cDNA template, and 3 μL of DNase-free H_2_O. The thermal cycling protocol was as follows: initial denaturation at 95 °C for 30 s; 40 cycles of denaturation at 95 °C for 5 s and annealing/extension at 60 °C for 30 s (with fluorescence acquisition); followed by a melting curve analysis (95 °C for 5 s, 60 °C for 60 s, and a continuous ramp to 95 °C with fluorescence measurement) and a final cooling step to 50 °C for 30 s. Gene expression levels were calculated using the 2^−ΔΔCt^ method, with the actin cytoskeleton gene serving as the internal reference.

### 2.8. Quantification of E. bromicola in E. bromicola–Hordeum Association

#### 2.8.1. Construction of the Standard Curve

The concentration of the linearized plasmid *perA* was measured five times using a Promega NanoDrop spectrophotometer (Promega Corporation, Madison, WI, USA), and the obtained values were converted to a logarithmic scale. Subsequently, the plasmid was serially diluted with DNase-free H_2_O to generate a standard curve spanning concentrations from 10^9^ to 10^3^ ng/µL. Amplification was performed on a Bio-Rad CFX96 Real-Time System (Bio-Rad Laboratories, Inc., Hercules, CA, USA) using a SYBR Green-based method under the following conditions: initial denaturation at 95 °C for 10 min, followed by 35 cycles of denaturation at 95 °C for 30 s, annealing at 60 °C for 30 s, and extension at 72 °C for 30 s. A melt-curve analysis was conducted at the end of each run by gradually increasing the temperature from 65 °C to 95 °C in 0.5 °C increments every 10 s, followed by a final step at 95 °C for 1 min. The standard curve was defined by the equation Y = −3.626X + 38.481, R^2^ = 0.995. In the resulting standard curve, X represents the logarithm of plasmid concentration (copies/µL), and Y represents the corresponding Ct value.

#### 2.8.2. Absolute Quantification of *E. bromicola* Biomass in *E. bromicola*–Hordeum Association

Genomic DNA was extracted from the three associations (NHb+Eb, Hb+Eb, Hv+Eb) at both seedling and tillering stages, as described in Section 2.5.1. Based on the standard curve established in Section 2.8.1, the mycelial biomass of *E. bromicola* in three associations was quantified by real-time PCR. Each 10 µL reaction contained 5 µL of 2× SYBR Green Master Mix, 0.5 µL each of forward and reverse primers (10 µM), 1 µL of DNA template (40 ng/µL), and 3 µL of sterile ddH_2_O. The thermal cycling protocol was as follows: initial denaturation at 95 °C for 10 min; 35 cycles of 95 °C for 30 s, 60 °C for 30 s, and 72 °C for 30 s; followed by a melting curve analysis from 65 °C to 95 °C with a ramp rate of 0.5 °C per 10 s, and a final hold at 95 °C for 1 min. All reactions were performed in three biological replicates per group, each with three technical replicates, along with a negative control in which the template was replaced with an equal volume of ddH_2_O. The obtained Ct values were converted to fungal copy numbers (copies ng^−1^ gDNA) using the established standard curve.

### 2.9. Statistical Analysis

Statistical analysis was performed using SPSS software (version 26.0; SPSS, Inc., Chicago, IL, USA). All data were assessed for normality (Shapiro–Wilk test) and homogeneity of variance (Levene’s test). The results indicated that, at the tillering stage, the assumptions of normality and equal variance were violated for the cell wall thickness data comparing *E. bromicola–Hordeum* associations (NHb+Eb, Hb+Eb, Hv+Eb) with their corresponding *E. bromicola*-free *Hordeum* (NHb, Hb, Hv) and the expression levels of *Nox A*, *Nox C*, and *Rac* genes in the *E. bromicola*–*Hordeum* association (NHb+Eb, Hb+Eb, Hv+Eb). At the seedling stage, the expression data of *Nox B* genes in the symbiotic combinations also failed to meet these parametric assumptions. Since the data violated the normality assumption, all continuous variables were subjected to log transformation [log(x + 1)]. The rest of the data were analyzed using the ANOVA. Fisher’s Least Significant Difference (LSD) test (*p* < 0.05) was conducted to assess the significance of differences among the means. In all tests, a *p* value < 0.05 was considered statistically significant. All measurements were shown as mean ± standard deviation.

## 3. Results

### 3.1. E. bromicola–Hordeum Association Was Successfully Constructed

**Figure 3:**
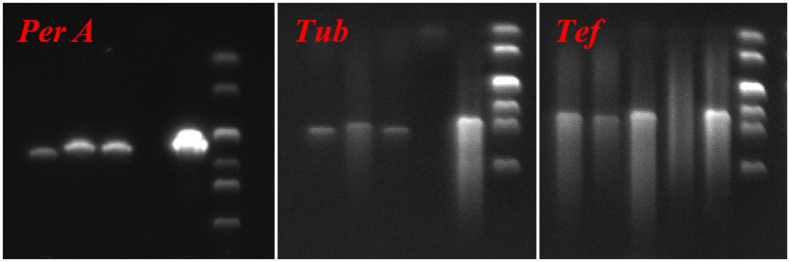
Verification of inoculation on wild barley and barley. Note: From left to right, each figure shows Hv+Eb (cultivated barley inoculated with *E. bromicola*), Hb+Eb (cultivated wild barley inoculated with *E. bromicola*), NHb+Eb (native wild barley naturally infected with *E. bromicola*), Negative, Positive.

The NHb+Eb, Hb+Eb, and Hv+Eb associations were amplified by PCR using *tefA*, *tubB*, and *PerA* gene fragments. The *E. bromicola* WBE1 strain was used as a positive control, and water was used as a negative control. The agarose gel electrophoresis was used for electrophoresis detection, and the test results were shown in Figure 3.

### 3.2. The Colonization of E. bromicola in the Leaf Sheath of Hordeum

**Figure 4:**
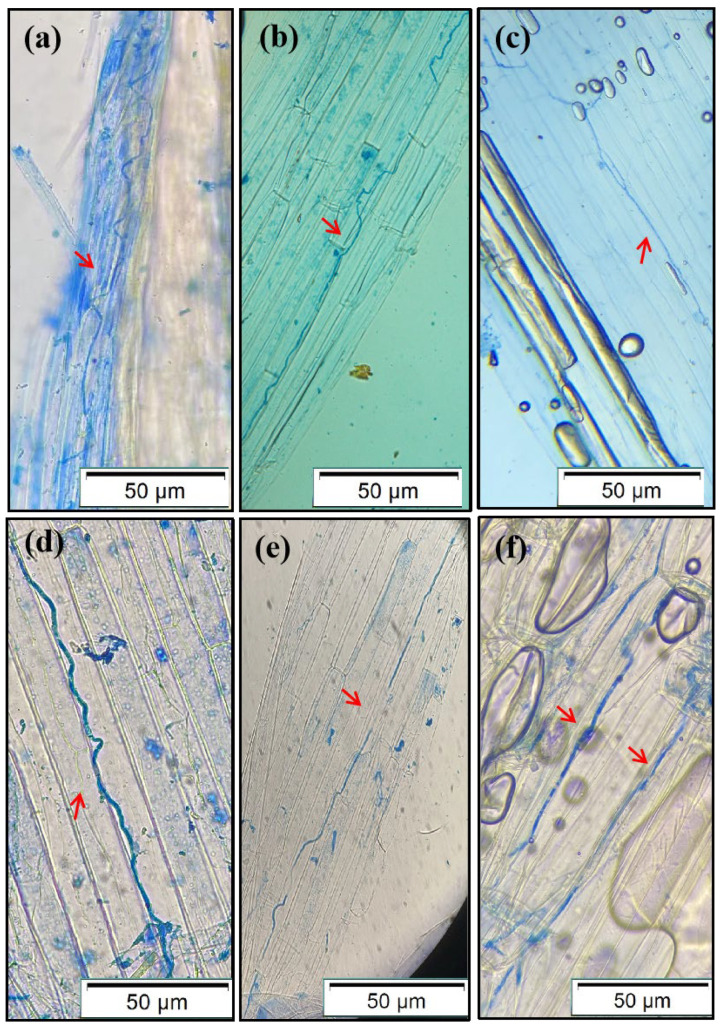
Aniline blue-stained mycelia in the leaf sheath of NHb+Eb, Hb+Eb, and Hv+Eb. Note: (**a**) NHb+Eb (native wild barley naturally infected with *E. bromicola* of young leaves at seedling stage), (**b**) Hb+Eb (cultivated wild barley inoculated with *E. bromicola* of young leaves at seedling stage), (**c**) Hv+Eb (cultivated barley inoculated with *E. bromicola* of young leaves at seedling stage), (**d**) NHb+Eb (native wild barley naturally infected with *E. bromicola* of mature leaves at tillering stage), (**e**) Hb+Eb (cultivated wild barley inoculated with *E. bromicola* of mature leaves at tillering stage), (**f**) Hv+Eb (cultivated barley inoculated with *E. bromicola* of young leaves at tillering stage). The structures indicated by the red arrows are the mycelia.

The hyphae of *E. bromicola* extended longitudinally along the NHb+Eb, Hb+Eb, and Hv+Eb cell walls, parallel to the cell axis at the seedling stage and tillering stage (Figure 4). The hyphae of NHb+Eb and Hb+Eb curved without branches (Figure 4a,b,d,e), but the association of Hv+Eb hyphae had branches and no bending (Figure 4c,f). The hyphae of NHb+Eb at the tillering stage were thicker than those at the seedling stage (Figure 4a,d). Compared with NHb+Eb mycelium, the middle of Hb+Eb mycelium was broken (Figure 4b,e). The mycelium of the Hv+Eb association at the tillering stage was thicker and shorter than that at the seedling stage (Figure 4c,f).

**Figure 5:**
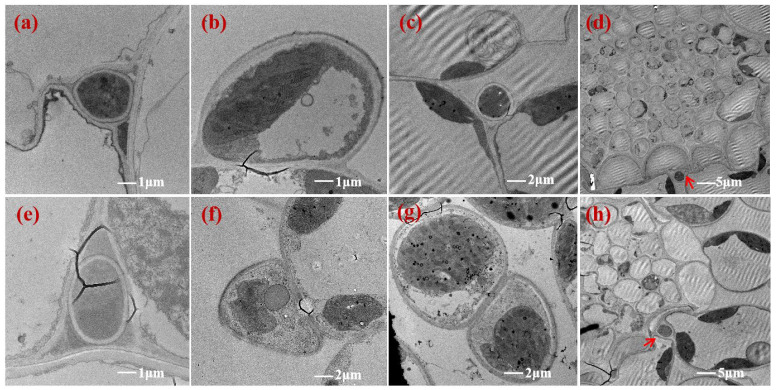
Transmission electron microscopy (TEM) images showing the ultrastructure of *E. bromicola* in leaves of NHb+Eb. Note: (**a**–**d**) NHb+Eb at seedling stage; (**e**–**h**) NHb+Eb at tillering stage. NHb+Eb: native wild barley naturally infected with *E. bromicola*. The structures indicated by the red arrows are the mycelia.

**Figure 6:**
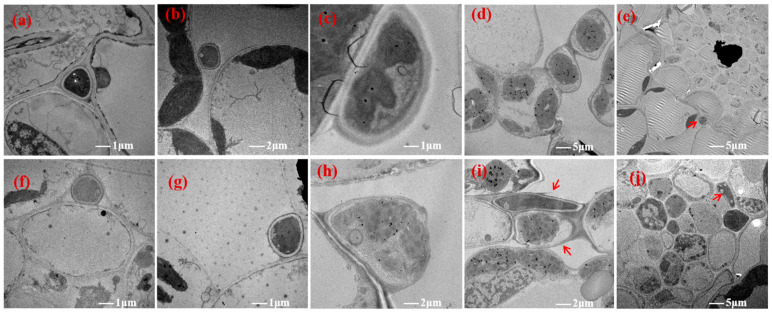
Transmission electron microscopy (TEM) images showing the ultrastructure of *E. bromicola* in leaves of Hb+Eb. Note: (**a**–**e**) Hb+Eb at seedling stage; (**f**–**j**) Hb+Eb at tillering stage. Hb+Eb: cultivated wild barley inoculated with *E. bromicola*. The structures indicated by the red arrows are the mycelia.

Transmission electron microscopy showed that the hyphae were flat (Figure 5b,f and Figure 6c,h), wedge-shaped (Figure 5a,e and Figure 6a,e), or stretched (Figure 5c and Figure 6b,g) among the NHb+Eb and Hb+Eb cell walls, indicating that the hyphae were firmly attached to the NHb+Eb and Hb+Eb cell walls. At the contact point between the *E. bromicola* and the NHb+Eb and Hb+Eb, there are small dense cytoplasm in the cell wall of the host (Figure 5b,f and Figure 6h). The hyphae of NHb+Eb and Hb+Eb were in direct contact with the outer surface of the sheath cells around the vascular bundle tissues (Figure 5d,h and Figure 6e,j). The hyphae cells of NHb+Eb appeared to have a fusion phenomenon at the tillering stage (Figure 5g). However, the hyphae cells of Hb+Eb appeared to have a fusion phenomenon at the seedling stage and tillering stage (Figure 6d,i), and the mycelium appeared to have an irregular shape at the tillering stage (Figure 6i,j).

**Figure 7:**
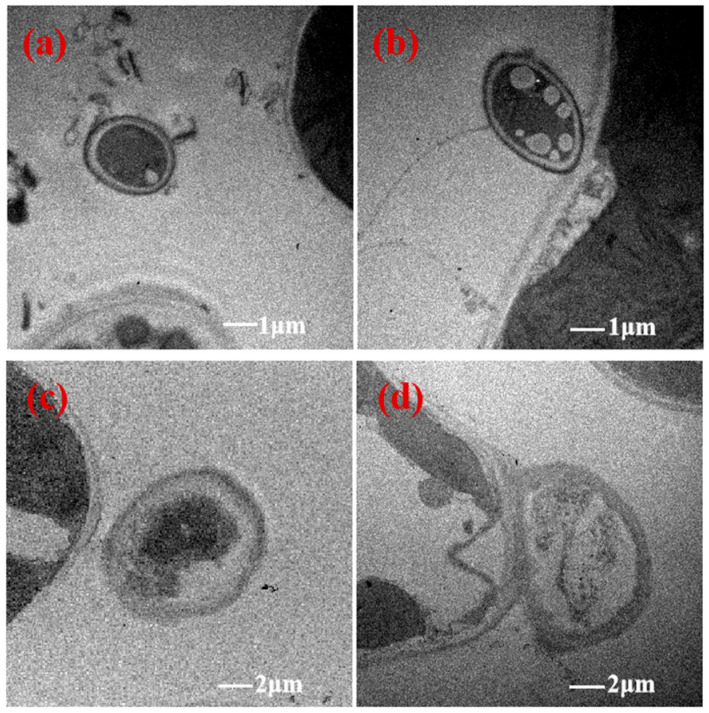
Transmission electron microscopy (TEM) images showing the ultrastructure of *E. bromicola* in leaves of Hv+Eb. Note: (**a**,**c**) Hv+Eb at seedling stage; (**b**,**d**) Hv+Eb at tillering stage. Hv+Eb: cultivated barley inoculated with *E. bromicola*.

The hyphae in mesophyll cells were far away from the cell wall of the Hv+Eb association (Figure 7a,c). The hyphae were flat between Hv+Eb cells at the tillering stage (Figure 7d). The hyphae were seriously vacuolated (Figure 4b,d, Figure 5b,d, Figure 6b,d, and Figure 7b,d). The chloroplasts were often missing near the contact point of hyphae with NHb+Eb, Hb+Eb, and Hv+Eb cell walls (Figure 5c,e,f, Figure 6f,g,h, and Figure 7b,d).

### 3.3. The Mycelial Diameter in Leaves of the E. bromicola–Hordeum Association

**Figure 8:**
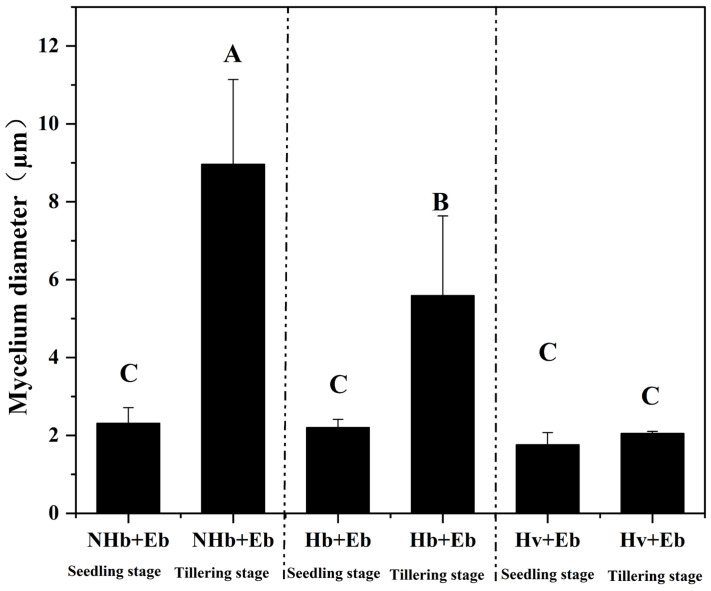
Diameters of *E. bromicola* in leaves of NHb+Eb, Hb+Eb, and Hv+Eb. Note: native wild barley naturally infected with *E. bromicola*; Hb+Eb: cultivated wild barley inoculated with *E. bromicola*; Hv+Eb: cultivated barley inoculated with *E. bromicola*. Different uppercase letters mean significance among different host plants in the different growth periods using the LSD test at *p* < 0.05, *n* = 5.

Transmission electron microscopy revealed that both the growth period and barley species affected the mycelial diameter of *E. bromicola*. Therefore, based on Figure 5, Figure 6 and Figure 7, we quantified the diameter of *E. bromicola* in NHb+Eb, Hb+Eb, and Hv+Eb leaves at the seedling stage and tillering stage. The results showed that at the tillering stage, the mycelial diameters in the NHb+Eb and Hb+Eb treatment groups were significantly (*p* > 0.05) larger than those in their corresponding treatment groups at the seedling stage. However, there was no significant difference in mycelial diameter between the seedling and tillering stages of Hv+Eb (*p* > 0.05). There was no significant difference (*p* > 0.05) in mycelial diameter between NHb+Eb, Hb+Eb, and Hv+Eb at the seedling stage. The mycelial diameter of NHb+Eb was significantly (*p* < 0.05) larger than that of Hb+Eb and Hv+Eb at the tillering stage (Figure 8).

### 3.4. Effects of E. bromicola on the Cell Wall of NHb, Hb, and Hv

**Figure 9:**
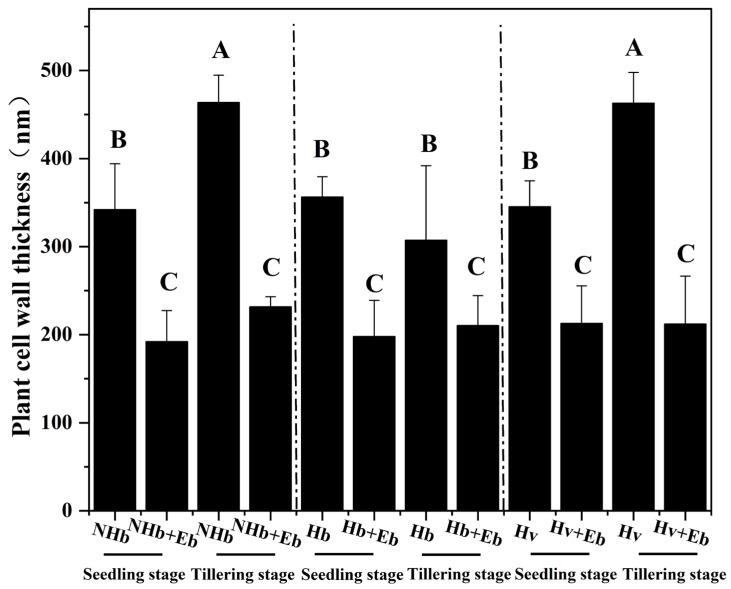
Effects of *E. bromicola* on the cell wall of NHb+Eb, Hb+Eb, and Hv+Eb. Note: NHb+Eb: native wild barley naturally infected with *E. bromicola*; NHb: native wild barley uninfected with *E. bromicola*; Hb+Eb: cultivated wild barley inoculated with *E. bromicola*; Hb: cultivated wild barley uninoculated with *E. bromicola*; Hv+Eb: cultivated barley inoculated with *E. bromicola*; Hv: cultivated barley uninoculated with *E. bromicola*. Different uppercase letters mean significance among different host plants in the different growth periods using the LSD test at *p* < 0.05, *n* = 5.

Transmission electron microscopy revealed that *E. bromicola* affected the cell wall thickness of three barley. Therefore, based on Figure 5, Figure 6 and Figure 7, we quantified the cell wall thickness of three barley. At both the seedling and tillering stages, the cell walls of NHb+Eb, Hb+Eb, and Hv+Eb were significantly thicker than those of NHb, Hb, and Hv, respectively (Figure 9). It indicates that colonization by *E. bromicola* reduces host cell wall thickness.

### 3.5. Effects of E. bromicola on NHb and Hb Trichomes

**Figure 10:**
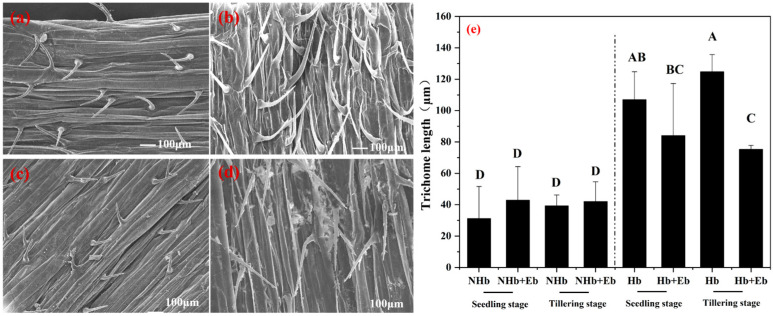
Scanning electron microscopy (SEM) images showing the effects of *E. bromicola* on the trichome of NHb and Hb. Note: (**a**) NHb+Eb at tillering stage, (**b**) Hb+Eb at tillering stage, (**c**) NHb at tillering stage, (**d**) Hb at tillering stage, (**e**) Length of trichome of NHb+Eb vs. NHb and Hb+Eb vs. Hb at different growth stages. NHb+Eb: native wild barley naturally infected with *E. bromicola*; NHb: native wild barley uninfected with *E. bromicola*; Hb+Eb: cultivated wild barley inoculated with *E. bromicola*; Hb: cultivated wild barley uninoculated with *E. bromicola*. Different uppercase letters mean significance among different host plants in the different growth periods using the LSD test at *p* < 0.05, *n* = 5.

Scanning electron microscopy revealed that *E. bromicola* affected the trichomes of barley (NHb and Hb). Therefore, we measured the size of the trichome. The results showed that *E. bromicola* had no significant effect on the trichomes of NHb host plants (*p* > 0.05). However, the length of Hb+Eb trichomes was significantly (*p* < 0.05) shorter than that of Hb at the tillering stage (Figure 10).

### 3.6. Effect of E. bromicola on Hv Stomata

**Figure 11:**
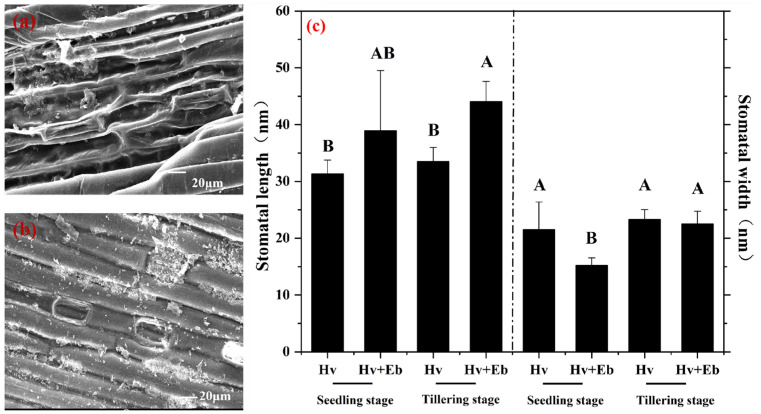
Scanning electron microscopy (SEM) images showing the effects of *E. bromicola* on the stomata of Hv+Eb. Note: (**a**) Hv+Eb at seedling stage, (**b**) Hv+Eb at tillering stage. (**c**) The stomatal length and width at seedling stage and tillering stage. Hv+Eb: cultivated barley inoculated with *E. bromicola*; Hv: cultivated barley uninoculated with *E. bromicola*. Different uppercase letters mean significance among different host plants in the different growth periods using the LSD test at *p* < 0.05, *n* = 5.

The hyphae in leaves of NHb, Hb, and Hv at different growth stages were examined by scanning electron microscopy. It was found that *E. bromicola* affected the Hv stomata. Therefore, we measured the size of the stomata. The width of Hv was significantly (*p* < 0.05) larger than that of Hv+Eb at the seedling stage (Figure 11). It indicates that colonization by *E. bromicola* results in stomatal closure of Hv.

### 3.7. The Differential Gene Expression

#### 3.7.1. The RT-PCR Analysis of Iron Regulatory Genes

**Figure 12:**
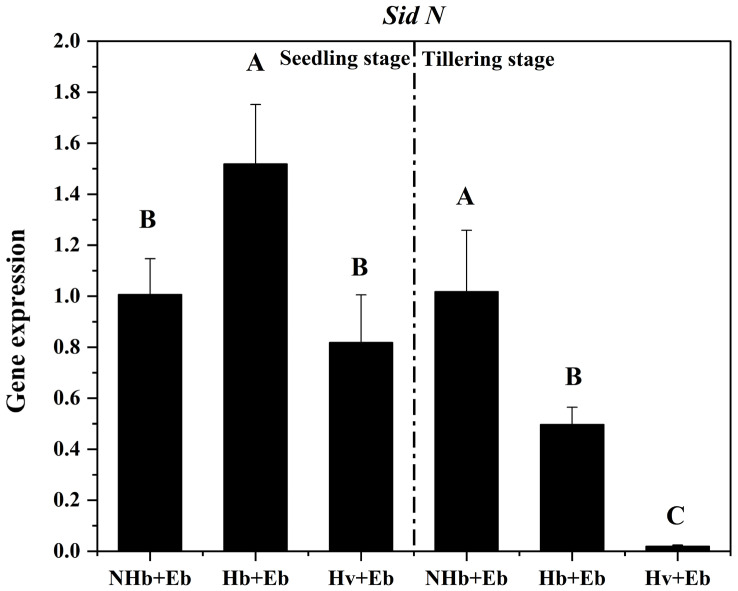
Expression of siderophore genes in NHb+Eb, Hb+Eb, and Hv+Eb. Note: NHb+Eb: native wild barley naturally infected with *E. bromicola*; Hb+Eb: cultivated wild barley inoculated with *E. bromicola*; Hv+Eb: cultivated barley inoculated with *E. bromicola*. Different uppercase letters mean significance among different host plants in the different growth periods using the LSD test at *p* < 0.05, *n* = 3.

We obtained six symbiotic genes by gene cloning. At the seedling stage, Hb+Eb *SidN* expression was significantly higher than in NHb+Eb (*p* < 0.05). At the tillering stage, the expression of both Hb+Eb *SidN* and Hv+Eb *SidN* was significantly lower than in NHb+Eb (*p* < 0.05) (Figure 12).

#### 3.7.2. The RT-PCR Analysis of Genes Regulating Hyphal Fusion

**Figure 13:**
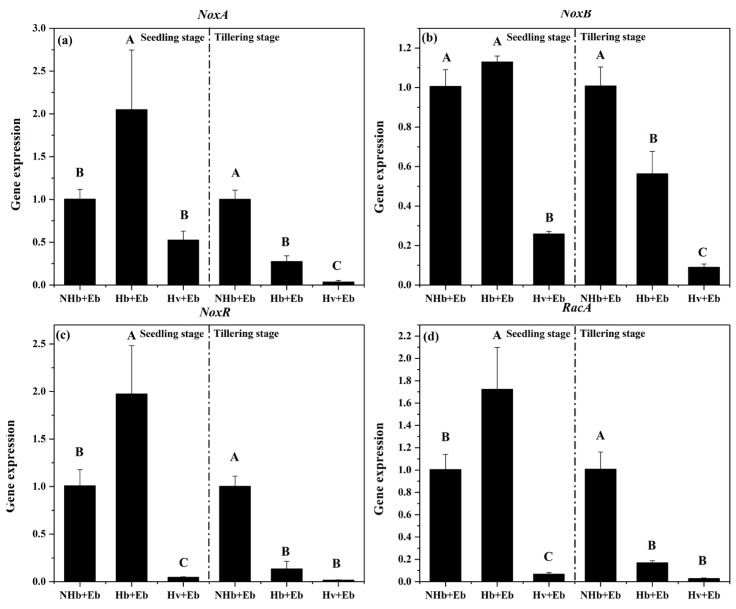
Expression of *NoxA* (**a**), *NoxB* (**b**) *NoxR* (**c**) and *RacA* (**d**) in NHb+Eb, Hb+Eb, and Hv+Eb at seedling stage and tillering stage. Note: NHb+Eb: native wild barley naturally infected with *E. bromicola*; Hb+Eb: cultivated wild barley inoculated with *E. bromicola*; Hv+Eb: cultivated barley inoculated with *E. bromicola*. Different uppercase letters mean significance among different host plants in the different growth periods using the LSD test at *p* < 0.05, *n* = 3.

At the seedling stage, Hb+Eb *NoxA* expression was significantly higher than in NHb+Eb (*p* < 0.05). However, at the tillering stage, the expression of both Hb+Eb *NoxA* and Hv+Eb *NoxA* was significantly lower than in NHb+Eb (*p* < 0.05) (Figure 13a).

At the seedling stage, Hv+Eb *NoxB* expression was significantly lower than in NHb+Eb (*p* < 0.05). At the tillering stage, the expression of both Hb+Eb *NoxB* and Hv+Eb *NoxB* was significantly lower than in NHb+Eb (*p* < 0.05) (Figure 13b).

At the seedling stage, the expression of *NoxR* and *RacA* in Hb+Eb was significantly higher than in NHb+Eb (*p* < 0.05), whereas the expression of *NoxR* and *RacA* in Hv+Eb was significantly lower (*p* < 0.05) (Figure 13c,d). At the tillering stage, the expression of both genes in Hb+Eb and Hv+Eb was significantly lower than in NHb+Eb (*p* < 0.05) (Figure 13c,d).

#### 3.7.3. The Mycelial Concentration of *E. bromicola* –Hordeum Association Leaves

**Figure 14:**
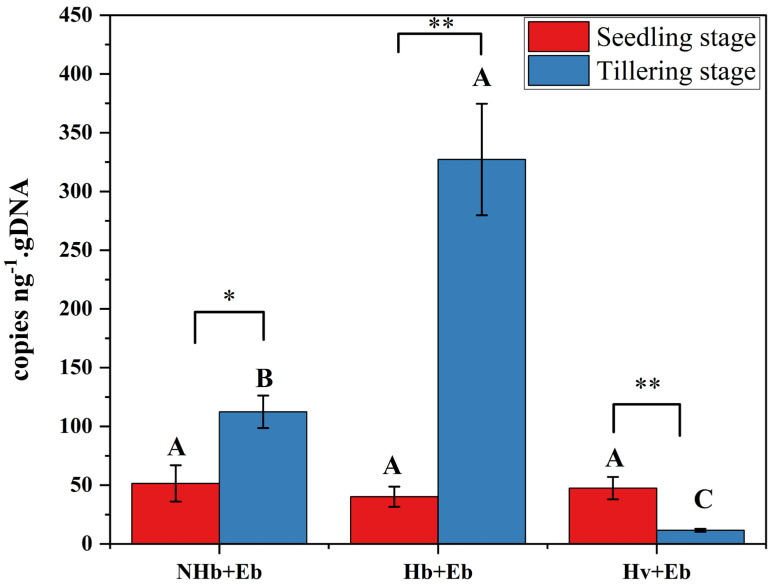
The mycelial content of the *E. bromicola*–*Hordeum* association. Note: NHb+Eb: native wild barley naturally infected with *E. bromicola*; Hb+Eb: cultivated wild barley inoculated with *E. bromicola*; Hv+Eb: cultivated barley inoculated with *E. bromicola*. The different uppercase letters mean significance among different symbiont leaves in the same growth period using the LSD test at *p* < 0.05. * and ** indicate significant differences at *p* < 0.05 and *p* < 0.001, *n* = 3, respectively, between the seedling and tillering stages.

At the seedling stage, there were no significant differences in leaf mycelial concentration among NHb+Eb, Hb+Eb, and Hv+Eb. In contrast, at the tillering stage, the concentration in Hv+Eb was significantly lower than in both NHb+Eb and Hb+Eb (*p* < 0.05). Furthermore, from the seedling to the tillering stage, mycelial concentration significantly increased in NHb+Eb (*p* < 0.05) and Hb+Eb (*p* < 0.001) but significantly decreased in Hv+Eb (*p* < 0.001) (Figure 14).

## 4. Discussion

### 4.1. Effects of the Host Genotype on the Compatibility of the E. bromicola–Hordeum Association

Forage shortage in China restricts the sustainable development of grassland animal husbandry. The construction of symbiotic associations using the *Epichloë* endophyte, which produces only the insect-deterrent alkaloid peramine, provides a sustainable strategy for breeding high-quality, stress-tolerant forage grasses. The early work by our research group revealed that when *E. bromicola* isolated from *H. brevisubulatum* was inoculated into the cultivated barley cultivars ‘Yangsimai No. 1’ and ‘Chaiqing No. 1’, it successfully created the *Epichloë bromicola*–barley germplasm [6]. To further explore the potential of this germplasm, we conducted a detailed investigation into the compatibility between *E. bromicola* and cultivated barley. This study demonstrates that the hyphae of *E. bromicola* were longitudinally arranged along the leaf axis in the symbiont tissues of NHb+Eb (native wild barley naturally infected with *E. bromicola*), Hb+Eb (cultivated wild barley inoculated with *E. bromicola*), and Hv+Eb (cultivated barley inoculated with *E. bromicola*). This is the characteristic of the synchronous growth of hyphae and novel host [1]. However, it was found by transmission electron microscopy that the mycelium of *E. bromiicola* was flat, wedge-shaped, or stretched between the cell walls of NHb+Eb and Hb+Eb leaves, while it was only flat between the cell walls of the Hv+Eb association. The results demonstrate that both the diversity of contact modes and the contact area between *E. bromicola* hyphae and the host cell wall are significantly smaller in the Hv+Eb compared to those of NHb+Eb and Hb+Eb. *Epichloë* endophytes are obligate grass endophytes. If they want to successfully colonize and survive in the host intercellular space (apoplast), they must rely on the host plant apoplast nutrients [14]. The reduced contact area and simplified interaction morphology in Hv+Eb likely impair the efficiency of nutrient acquisition by the fungus. This notion is supported by the observed cytoplasmic dispersion in Hv+Eb hyphae. We propose that impaired nutrient acquisition may be a primary factor leading to stunted hyphal development in the Hv+Eb association. Previous studies on nutrient utilization in endophyte–grass systems have yielded diverse results. While Christensen et al. (2002) [14] identified sucrose as a key nutrient for *Neotyphodium* endophytes, White et al. (1993) [33] suggested arabinose and xylose are primary carbon sources for *Acremonium* endophytes. Our preliminary measurements of sucrose content in the different associations showed irregular patterns, suggesting that *E. bromicola* may utilize a different suite of sugars, highlighting a species- or strain-specific nutrient strategy. Future research should profile a broader range of apoplastic carbohydrates to elucidate the link between host-derived nutrients and hyphal growth in association.

Critically, Christensen et al. (2012) [34] emphasized that cytoplasmic dispersion and vacuolization serve as characteristic markers of symbiotic dysfunction in *Epichloë* endophytes, often associated with uncontrolled hyphal proliferation, hyphal degeneration, and impaired host development. The *E. bromicola* did not unrestrictedly grow in the Hv+Eb association. Moreover, with the extension of the growth period, the diameters of NHb+Eb and Hb+Eb hyphae increase, but the hyphae diameters in the Hv+Eb association have no change. The results of mycelium concentration and mycelium diameter also complement each other. The above results demonstrate that the compatibility problem of Hv+Eb is linked to hyphae vacuolization. However, the physiological and molecular mechanism of hyphal vacuolization is still unclear.

The differential outcomes across NHb+Eb, Hb+Eb, and Hv+Eb underscore that symbiotic compatibility is profoundly shaped by host genotype [35]. The differential responses of hosts to Epichloë symbionts, ranging from tolerance to rejection, are closely linked to host genetic evolution. In natural associations such as *E. bromicola* with its native wild barley (*H. brevisubulatum*), long-term coevolution has likely fine-tuned reciprocal recognition, nutrient exchange, and immune modulation, enabling the fungus to establish a balanced and persistent mutualism. In contrast, the incompatibility manifested in Hv+Eb may arise from the absence of such co-adaptive traits in the cultivated barley genome, which has been selected primarily for agronomic yield rather than symbiotic competence. This insight provides a critical theoretical basis for the rational selection of host-endophyte pairs in breeding programs. Therefore, future research should focus on the genetic basis of both the host plant and the *Epichloë* fungus to further elucidate their roles in shaping the establishment mechanisms of cereal crop–endophyte symbiotic associations.

### 4.2. Effects of E. bromicola on the Cell Morphology of the Host

Endophytic fungi can shape symbiotic outcomes by inducing a range of biochemical, morphological, and physiological changes in their host plants [36]. The morphological features can offset the adverse effects of the environment [37]. For instance, trichome development in *Arabidopsis thaliana* is regulated by gibberellins [38], and inoculation with *Epichloë festucae* was shown to upregulate gibberellin biosynthetic genes in perennial ryegrass [39]. It is speculated that the upregulated expression of gibberellin signaling may be a response to *Epichloë* endophyte infection, potentially influencing trichome development. Our findings partially support this hypothesis, as *E. bromicola* colonization altered trichome morphology in the Hb+Eb association. However, contrary to expectations of enlargement, we observed a reduction in trichome size, highlighting that the direction of morphological change is likely contingent on the specific host–endophyte genotype combination. Enlarged trichomes and stomatal closure are often associated with improved water retention in endophyte-infected plants [39]. Wang et al. (2021) reported that the Hv+Eb association exhibited enhanced tolerance to both drought and waterlogging [40]. In line with this, we observed *E. bromicola*-induced stomatal closure in Hv+Eb plants at the tillering stage. These findings indicate that tolerance to both drought and waterlogging in the Hv+Eb association is conferred by *E. bromicola* via modification of leaf structure.

### 4.3. Disrupted Iron Homeostasis and Redox Signaling Are Associated with Hyphal Vacuolization

Iron is indispensable for fundamental cellular processes across nearly all organisms, including respiration, DNA synthesis, and oxidative stress management [41]. In fungal endophytes like *Epichloë festucae*, siderophore-mediated iron acquisition is vital for maintaining iron homeostasis, successful *E. festucae* colonization, and mutualistic persistence [15,16,17]. In this study, expression of *sidN*, a key gene in siderophore biosynthesis, was significantly lower in the Hv+Eb compared to both Hb+Eb and NHb+Eb. This downregulation likely compromises the production of extracellular siderophores (e.g., *epichloënin A*), limiting iron acquisition from the host apoplast [16]. The iron deficiency that consequently occurs in the Hv+Eb association is potentially associated with hyphal vacuolization and growth restriction.

Furthermore, iron homeostasis is intricately linked to fungal oxidative stress tolerance [42,43,44]. The maintenance of a balanced mutualism requires precise spatiotemporal regulation of fungal reactive oxygen species (ROS) signaling. Johnson et al. (2013) [7] demonstrated that disrupting fungal ROS regulation collapses the symbiosis. Their work further suggested that under iron limitation, *E. festucae* preferentially utilizes the extracellular siderophore *epichloënin A* (EA) over the intracellular ferricrocin (FC) to mitigate oxidative stress [15]. In our study, transcriptional analysis revealed significant downregulation of genes encoding the NADPH oxidase (*Nox*) complex in the Hv+Eb, implying a disruption in redox signaling. While this suggests a link between impaired iron acquisition, altered ROS signaling, and symbiotic failure, whether *Nox* downregulation directly causes hyphal vacuolization remains correlative. Future investigations should directly quantify iron levels, measure catalase/peroxidase activities, and visualize ROS fluxes to delineate the causal interplay between iron homeostasis, oxidative stress, and hyphal integrity in these symbiotic systems.

In the incompatible Hv+Eb association observed in this study, the downregulation of *sidN* and the Nox complex genes aligns with the well-established roles of iron acquisition and reactive oxygen species (ROS) signaling in maintaining mutualistic symbiosis, as previously demonstrated in the *E. festucae*–*Lolium perenne* model system [15,22,24]. However, the phenotypic outcomes markedly differ between the two systems. In *E. festucae*, disruption of siderophore biosynthesis, such as in the ΔsidN mutant, typically induces hyperbranching, hyphal curling, and swelling [7]. In contrast, in our Hv+Eb, similar molecular perturbations result in pronounced hyphal vacuolization accompanied by growth restriction. This comparison suggests that although the core genetic pathways regulating symbiotic compatibility may be conserved within the *Epichloë* genus, the specific manifestations of incompatibility are highly dependent on the particular host–endophyte genotype combination. Therefore, the hyphal vacuolization reported here likely represents a distinct incompatibility response of *E. bromicola* when confronted with the apoplastic environment of a cultivated cereal host. This finding underscores that mechanistic insights derived from model symbiotic systems cannot be directly extrapolated to other symbiotic contexts and must be validated within specific experimental systems.

Based on these findings, this study proposes several feasible strategies to enhance the colonization potential of endophytic fungi in non-natural hosts. First, improving fungal iron acquisition capacity, for example by overexpressing fungal genes such as *sidN*, could help overcome iron limitation in the host apoplast. Second, modulating host iron homeostasis through genetic engineering, such as by increasing apoplastic iron availability or expressing plant-derived iron chelators, may create a more favorable niche for fungal colonization. Third, screening or breeding host genotypes capable of sustaining high expression of fungal *Nox* complex genes could help maintain redox balance and promote symbiotic stability. These approaches may mitigate hyphal vacuolization and foster more robust symbiotic interactions. In summary, molecular insights into iron uptake and redox signaling pathways can provide a theoretical basis for the targeted development of more compatible cereal–*Epichloë* symbiotic systems for agricultural applications.

## 5. Conclusions

In conclusion, this study shows that the compatibility issue between *E. bromicola* and cultivated barley appears at the cellular level as hyphal vacuolization. At the gene expression level, this problem is linked to the downregulation of key genes involved in iron uptake (*sidN*) and symbiotic regulation (*Nox* complex). These results provide important phenotypic and molecular insights into the colonization barrier of *E. bromicola* in its non-natural host, cultivated barley. Further research should focus on elucidating the molecular mechanisms underlying this compatibility, which will help establish a theoretical basis for developing a stable and heritable cultivated barley inoculated with the *E. bromicola* symbiotic association.

## Figures and Tables

**Table 1:** The universal primer sources and sequence.

Number	Gene Name	Primer Name	Sequence (5′-3′)	Length	Purpose	Literature	
1	*NoxB*	*NoxB*-F	GCCACTTACACGAATGCTCCTCTC	374 bp	PCR	(Tanaka et al., 2006 [22])	AB236861.1
		*NoxB*-R	CGGTGGTGATGTGGAAGGTGATG				
2	*NoxA*	*noxA*f	CTTCAGAACCGCGACTAGCA	344 bp	PCR		
		*noxA*r	TGCAGAGGAGCATGACATGT				
3	*Sid N*	*Sid N*-F	ACGGAGGTTACGATTGGATGCA	245 bp	PCR	(Johnson et al., 2013 [7])	JN132404.1
		*Sid N*-R	TCGCCGGTCTTATACATCTTTC				
4	*RacA*	*RacA*-F	ATGGCTCAACCCGGTGTTCAGTC	600 bp	PCR	(Takemoto et al., 2007) [31]	AB260937
		*RacA*-R	TTACAGGATTGAACACTTGGACTTC				
5	*NoxR*	*noxR*-F	GTGCCGCAGTGAGCATCCTTGAAAC	515 bp	PCR		AB260938
		*noxR*-R	TCACAACATGCCTTTGCGGATCTC				
		*Ste12*-R	CTGGCCCATGTTGTTGTTGG				
6	*perA*	*perA*2-F	CGTCGTGGTAACGCACGCAAACG	651 bp	PCR	(Berry et al., 2015) [32]	
		*perA*2-R	CAGTCTGCCTTGCCGACgCGGGGT				

**Table 2:** Specific primer.

Number	Gene Name	Primer Name	Sequence (5′-3′)	Length	Purpose
1	*NoxB*	*NoxB*-F	AGAGCATCCGCCTTGGTCTTG	100 bp	QPCR
		*NoxB*-R	TGCCGTTGAGTGGTGTCTGG		
2	*NoxA*	*noxA*f	ATGATTGTACGGCACACGGTAGAAG	106 bp	QPCR
		*noxA*r	GCGACTAGCAGGGCTCAACAC		
3	*Sid N*	*Sid N*-F	CTTTCGCATCGGGTCTGTTCAG	72 bp	QPCR
		*Sid N*-R	GCATGGCAGGTGAGCTTGTTATC		
4	*RacA*	*RacA*-F	ATCAGTCTTGGATTGTGGGATACGG	108 bp	QPCR
		*RacA*-R	GGAGACGATGGAGAAGCAGATGAG		
5	*NoxR*	*noxR*-F	CTGACGAAGCAAGACGAGACAAG	148 bp	QPCR
		*noxR*-R	CATCTTTGCGTTCTTCCCACAATG		
6	*perA*	*perA*2-F	TGAGATAGCCTCGACCGACAAG	136 bp	QPCR
		*perA*2-R	ATGGCGACACACATTGGGAAAG		

## Data Availability

The original contributions presented in this study are included in the article. Further inquiries can be directed to the corresponding author.

## References

[1] Christensen M.J., Bennett R.J., Ansari H.A., Koga H., Johnson R.D., Bryan G.T., Simpson W.R., Koolaard J.P., Nickless E.M., Voisey C.R. (2008). *Epichloë* endophytes grow by intercalary hyphal extension in elongating grass leaves. Fungal Genet. Biol..

[2] Zabalgogeazcoa Í., Ciudad A.G., Aldana V.D., Criado B.G. (2006). Effects of the infection by the fungal endophyte *Epichloë* festucae in the growth and nutrient content of *Festuca rubra*. Eur. J. Agron..

[3] Schardl C.L., Young C.A., Hesse U., Amyotte S.G., Andreeva K., Calie P.J., Fleetwood D.J., Haws D.C., Moore N., Oeser B. (2013). Plant-symbiotic fungi as chemical engineers: Multi-genome analysis of the Clavicipitaceae reveals dynamics of alkaloid loci. PLoS Genet..

[4] Young C.A., Hume D.E., McCulley R.L. (2013). Forages and pastures symposium: Fungal endophytes of tall fescue and perennial ryegrass: Pasture friend or foe?. J. Anim. Sci..

[5] Liu J., Wang Z.F., Malik K., Li C.J. (2025). Vertically transmitted *Epichloë bromicola* has transgenerational positive effects in cultivated barley. Plant Soil.

[6] Li C.J., Wang Z.F., Chen T.X., Nan Z.B. (2022). Creation of novel barley germplasm using an *Epichloë* endophyte. Chin. Sci. Bull..

[7] Johnson L.J., de Bonth A.C.M., Briggs L.R., Caradus J.R., Finch S.C., Fleetwood D.J., Fletcher L.R., Hume D.E., Johnson R.D., Card S.D. (2013). The exploitation of epichloae endophytes for agricultural benefit. Fungal Divers..

[8] Simpson W.R., Faville M.J., Moraga R.A., Williams W.M., Mcmanus M.T., Johnson R.D. (2014). *Epichloë* fungal endophytes and the formation of synthetic symbioses in Hordeeae (=Triticeae) grasses. J. Syst. Evol..

[9] Simpson W.R., Tsujimoto H., Hume D.E., Johnson R.D. (2024). Alien chromatin from Hordeeae grasses enhances the compatibility of *Epichloë* endophyte symbiosis with the hexaploid wheat *Triticum aestivum*. J. Fungi.

[10] White J.F., Torres M.S. (2009). Defensive Mutualism in Microbial Symbiosis.

[11] Koga H., Christensen M.J., Bennett R.J. (1993). Incompatibility of some grass/*Acremonium* endophyte associations. Mycol. Res..

[12] Tan Y.Y., Spiering M.J., Scott V., Lane G.A., Christensen M.J., Schmid J. (2001). In planta regulation of extension of an endophytic fungus and maintenance of high metabolic rates in its mycelium in the absence of apical extension. Appl. Environ. Microbiol..

[13] Christensen M.J., Bennett R.J., Schmid J. (2001). Vascular bundle colonisation by *Neotyphodium* endophytes in natural and novel associations with grasses. Mycol. Res..

[14] Christensen M.J., Bennett R.J., Schmid J. (2002). Growth of *Epichloë/Neotyphodium* and p-endophytes in leaves of grass hosts. Mycol. Res..

[15] Forester N.T., Lane G.A., Steringa M., Lamontb I.L., Johnsona L.J. (2018). Contrasting roles of fungal siderophores in maintaining iron homeostasis in *Epichloë festucae*. Fungal Genet. Biol..

[16] Zhang W., Forester N.T., Applegate E.R., Liu X.Q., Johnson L.J. (2023). High-affinity iron uptake is required for optimal *Epichloë festucae* colonization of *Lolium perenne* and seed transmission. Mol. Plant Pathol..

[17] Johnson L.J., Koulmanet A., Christensenal M., Lane L.J. (2013). An Extracellular Siderophore Is Required to Maintain the Mutualistic Interaction of *Epichloë festucae* with *Lolium perenne*. PLoS Pathogens.

[18] Kosman D.J. (2010). Molecular mechanisms of iron uptake in fungi. Mol. Microbiol..

[19] Schrettl M., Kim H.S., Eisendle M., Kragl C., Nierman W.C., Heinekamp T., Werner E.R., Jacobsen I., Illmer P., Yi H. (2008). *SreA* mediated iron regulation in *Aspergillus fumigatus*. Mol. Microbiol..

[20] Hsu P.C., Yang C.Y., Lan C.Y. (2011). Candida albicans *Hap43* is a repressor induced under low-iron conditions and is essential for iron-responsive transcriptional regulation and virulence. Eukaryot. Cell..

[21] Johnson L. (2008). Iron and siderophores in fungal-host interactions. Mycol. Res..

[22] Tanaka A., Christensen M.J., Takemoto D., Park P., Scott B. (2006). Reactive oxygen species play a role in regulating a fungus-perennial ryegrass mutualistic interaction. Plant Cell.

[23] Tanaka A., Takemoto D., Hyon G.S., Park P., Scott B. (2008). *NoxA* activation by the small GTPase *RacA* is required to maintain a mutualistic symbiotic association between *Epichloë festucae* and perennial ryegrass. Mol. Microbiol..

[24] Takemoto D., Kamakura S., Saikia S., Becker Y., Wrenn R., Tanaka A., Sumimoto H. (2011). Polarity proteins Bem1 and *Cdc24* are components of the filamentous fungal NADPH oxidase complex. Proc. Natl. Acad. Sci. USA.

[25] Chen T.X., Simpson W.R., Song Q.Y., Chen S.H., Li C.J., Ahmad R.Z. (2019). Identification of *Epichloë* endophytes associated with wild barley (*Hordeum brevisubulatum*) and characterisation of their alkaloid biosynthesis. N. Z. J. Agric. Res..

[26] Latch G.C.M., Christensen M.J. (1985). Artificial infection of grasses with endophytes. Ann. Appl. Biol..

[27] Moon C.D., Miles C.O., Järlfors U., Schardl C.L. (2002). The evolutionary origins of three new *Neotyphodium* endophyte species from grasses indigenous to the Southern Hemisphere. Mycologia.

[28] Moon C.D., Guillaumin J.J., Ravel C., Li C.J., Craven K.D., Schardl C.L. (2007). New *Neotyphodium* endophyte species from the grass tribes Stipeae and Meliceae. Mycologia.

[29] Rahnama M., Johnson R., Voisey C.R., Simpson W.R., Fleetwood D.J. (2018). The global regulatory protein *VelA* is required for symbiosis between the endophytic fungus *Epichloë festucae* and *Lolium perenne*. Mol. Plant-Microbe Interact..

[30] Venkatesh B.G., Perumal P., Muthu S., Pichai S., Narayan K.S., Malairaj S. (2018). Enhanced method for High Spatial Resolution surface imaging and analysis of fungal spores using Scanning Electron Microscopy. Sci. Rep..

[31] Takemoto D., Tanaka A., Scott B. (2007). NADPH oxidases in fungi: Diverse roles of reactive oxygen species in fungal cellular differentiation. Fungal Genet. Biol..

[32] Berry D., Takach J.E., Schardl C.L. (2015). Disparate independent genetic events disrupt the secondary metabolism gene *perA* in certain symbiotic *Epichloë* species. Appl. Environ. Microbiol..

[33] White J.F., Morgan-Jones G., Morrow A.C. (1993). Taxonomy, life cycle, reproduction and detection of *Acremonium* endophytes. Agric. Ecosyst. Environ..

[34] Christensen M.J., Saulsbury K., Simpson W.R. (2012). Conspicuous epiphytic growth of an interspecific hybrid *Neotyphodium* sp. endophyte on distorted host inflorescences. Fungal Biol..

[35] Hill N.S., Stringer W.C., Rottinghaus G.E., Belesky D.P., Parrott W.A., Pope D.D. (1990). Growth, morphological, and chemical component responses of tall fescue to *Acremonium coenophialum*. Crop. Sci..

[36] Morse L., Faeth S.H., Day T. (2007). *Neotyphodium* interactions with a wild grass are driven mainly by endophyte haplotype. Funct. Ecol..

[37] Evert R.F. (2006). Esau’s Plant Anatomy: Meristems, Cells, and Tissues of the Plant Body: Their Structure, Function, and Development.

[38] Perazza D., Vachon G., Herzog M. (1998). Gibberellins promote trichome formation by up-regulating GLABROUS1 in *Arabidopsis*. Plant Physiol..

[39] Dupont P.Y., Eaton C.J., Wargent J.J., Fechtner S., Solomon P., Schmid J., Day R.C., Scott B., Cox M.P. (2015). Fungal endophyte infection of ryegrass reprograms host metabolism and alters development. New Phytol..

[40] Wang Z.F. (2021). Novel Symbiont Creation of Epichloë Bromicola—Barley and Its Stress Tolerance Evaluation.

[41] Pandey S.S. (2023). The role of iron in phytopathogenic microbe–plant interactions: Insights into virulence and host immune response. Plants.

[42] Oide S., Moeder W., Krasnoff S., Gibson D., Haas H., Yoshioka Y., Turgeon B.G. (2006). *NPS6*, encoding a nonribosomal peptide synthetase involved in siderophore—Mediated iron metabolism, is a conserved virulence determinant of plant pathogenic ascomycetes. Plant Cell.

[43] Pujol-Carrion N., De La Torre-Ruiz M.A. (2010). Glutaredoxins *Grx4* and *Grx3* of Saccharomyces cerevisiae play a role in actin dynamics through their trx domains, which contributes to oxidative stress resistance. Appl. Environ. Microbiol..

[44] Roberts E. (2022). A review of the Plant Growth Promoting Fungal and Bacterial endophytes of Tall Fescue. Grass Res..

